# Characteristics and outcomes of adult Ethiopian patients enrolled in HIV care and treatment: a multi-clinic observational study

**DOI:** 10.1186/s12889-015-1776-4

**Published:** 2015-05-03

**Authors:** Zenebe Melaku, Matthew R Lamb, Chunhui Wang, Sileshi Lulseged, Tsigereda Gadisa, Solomon Ahmed, Zelalem Habtamu, Hailubeza Alemu, Tamrat Assefa, Elaine J Abrams

**Affiliations:** ICAP-Columbia University, Mailman School of Public Health, 722 West 168th Street, 13th floor, New York, NY 10032 USA; Department of Epidemiology, Mailman School of Public Health, Columbia University, New York, NY USA; Centers for Disease Control and Prevention, Addis Ababa, Ethiopia; Oromia Regional Health Bureau, Addis Ababa, Ethiopia; College of Physicians & Surgeons, Columbia University, New York, NY USA

**Keywords:** HIV care, ART initiation, Ethiopia, Sub-Saharan Africa, PEPFAR, CD4 cell count, Pre ART, ART scale-up

## Abstract

**Background:**

We describe trends in characteristics and outcomes among adults initiating HIV care and treatment in Ethiopia from 2006-2011.

**Methods:**

We conducted a retrospective longitudinal analysis of HIV-positive adults (≥15 years) enrolling at 56 Ethiopian health facilities from 2006–2011. We investigated trends over time in the proportion enrolling through provider-initiated counseling and testing (PITC), baseline CD4+ cell counts and WHO stage. Additionally, we assessed outcomes (recorded death, loss to follow-up (LTF), transfer, and total attrition (recorded death plus LTF)) before and after ART initiation. Kaplan-Meier techniques estimated cumulative incidence of these outcomes through 36 months after ART initiation. Factors associated with LTF and death after ART initiation were estimated using Hazard Ratios accounting for within-clinic correlation.

**Results:**

93,418 adults enrolled into HIV care; 53,300 (57%) initiated ART. The proportion enrolled through PITC increased from 27.6% (2006–2007) to 44.8% (2010–2011) (p < .0001). Concurrently, median enrollment CD4+ cell count increased from 158 to 208 cells/mm^3^ (p < .0001), and patients initiating ART with advanced WHO stage decreased from 56.6% (stage III) and 15.0% (IV) in 2006–2007 to 47.6% (stage III) and 8.5% (IV) in 2010–2011. Median CD4+ cell count at ART initiation remained stable over time. 24% of patients were LTF before ART initiation. Among those initiating ART, attrition was 30% after 36 months, with most occurring within the first 6 months. Recorded death after ART initiation was 6.4% and 9.2% at 6 and 36 months, respectively, and decreased over time. Younger age, male gender, never being married, no formal education, low CD4+ cell count, and advanced WHO stage were associated with increased LTF. Recorded death was lower among younger adults, females, married individuals, those with higher CD4+ cell counts and lower WHO stage at ART initiation.

**Conclusions:**

Over time, enrollment in HIV care through outpatient PITC increased and patients enrolled into HIV care at earlier disease stages across all HIV testing points. However, median CD4+ cell count at ART initiation remained steady. Pre- and post-ART attrition (particularly in the first 6 months) have remained major challenges in ensuring prompt ART initiation and retention on ART.

## Background

Ethiopia has experienced a rapid scale up of antiretroviral treatment (ART) since 2005, when free ART was introduced. Following decentralization of HIV services to health centers beginning in the second half of 2006, the program has rapidly expanded the provision of ART services to urban and rural populations throughout the country. Currently an estimated 800,000 individuals in Ethiopia are living with HIV [1] and based on the Ethiopian Demographic and Health survey, the estimated adult (15–59 years) HIV prevalence was 1.5% in 2011 [2]. The number of individuals that ever started ART increased from about 9,700 at the end of 2004 [3], to nearly 290,000 by 2012 [4], with an estimated 61% of individuals eligible for ART receiving treatment [4].

Trends over time and characteristics of the patient population seeking HIV care in Ethiopia have been under-reported in the literature compared with higher-prevalence countries in sub-Saharan Africa. Several reports have focused on individual hospitals or health centers in Ethiopia [5-9], using aggregate-level national reporting data [10-13], or sampling methodologies to obtain patient-level information on a subset of key outcomes [14]. However, large-scale multi-regional assessments of the adult population seeking HIV care using routinely-collected patient-level data in Ethiopia have not been reported.

Ethiopia is a relatively low HIV prevalence country which in 2007 embarked on an expansive national program of universal HIV counseling and testing [15] where all individuals attending a clinical setting for any reason are offered HIV testing as part of routine services, a unique situation among countries in sub-Saharan Africa. These guidelines recommended an “opt out” approach offering HIV testing to *all* patients accessing health-care. The effectiveness of the PITC approach in earlier identification, enrollment in care, and ART initiation of HIV positive patients has not been examined in Ethiopia. This approach differs from Voluntary Counseling and Testing, where individuals attend clinic specifically to receive an HIV test. Furthermore, despite successes in rolling out a large scale ART program, significant hurdles remain in starting all ART-eligible patients on treatment and in retaining them in care [12]. Late ART initiation despite eligibility and high rates of loss to follow-up (LTF) among patients after enrollment into care compromise the benefits to be gained from care and treatment programs [3,10-13]. While death rates remain high among patients initiating ART at advanced stages of disease, information on death and trends over time among patients in pre-ART care and those on ART is lacking.

In the present analysis, we aim to describe trends over time in enrollment of adults identified through PITC vs. VCT testing approaches, key demographic and clinical trends at enrollment into HIV care and at ART initiation, as well as outcomes after enrollment in care and ART initiation.

## Methods

### Study design and study population

We conducted an observational longitudinal analysis using routinely-collected clinical information on HIV-infected adults enrolled at 56 health facilities from 4 regions (Oromia, Dire Dawa, Harari, and Somali) in Ethiopia. These health facilities collected longitudinal electronic patient-level clinical information as part of routine care provision which were de-identified and included in the Optimal Models of HIV Care study. Data collection procedures for the Optimal Models study have been described elsewhere [16]. ICAP at Columbia University (ICAP) is an implementing partner supporting HIV care and treatment services in Ethiopia under funding from the President’s Emergency Plan for AIDS Relief (PEPFAR) through the US Centers for Disease Control and Prevention (CDC). At the end of 2011, ICAP was providing technical assistance to 86 hospitals and health centers, including 56 with electronic patient-level data that are included in this analysis. These 56 sites constitute approximately 90% of the data of all adults enrolled in HIV care at ICAP supported health facilities during the 2006-2011time period.

Information on all adult patients (≥15 years of age) enrolled in care between January 1, 2006 and December 31, 2011 were included in this analysis. Information on follow-up visits was included through December 31, 2012. CD4+ cell counts at enrollment into HIV care and ART initiation were taken as the most recent CD4+ cell count recorded within 3 months prior to one month after enrollment or ART initiation. Patient information routinely collected during each clinic visit was documented by health care providers on national patient forms and subsequently entered by trained data clerks into a patient-level database developed by ICAP. Data quality assessment was conducted quarterly to check completeness and accuracy. De-identified versions of electronic databases were compiled semi-annually by ICAP database managers and shared with study investigators at ICAP.

### Statistical methods

Descriptive statistics are presented on demographic and clinical characteristics of the study population overall, and stratified by year of enrollment into HIV care and, for those starting treatment, year of ART initiation. CD4+ cell count at enrollment and ART initiation were identified within a window period of three months prior, to one month after enrollment or ART initiation, respectively. Chi-square tests for group difference are presented for categorical variables in Tables 1 and 2. In addition, variables hypothesized a priori to change over time as a result of PITC scale-up (WHO stage and CD4+ cell count at enrollment into care/ART initiation, PITC and VCT point of entry) CD4 were tested for trends over time using univariate linear mixed models (ordinal outcomes for WHO stage and CD4 categories; binomial outcome for PITC and VCT) with a random effect to account for with-site correlations.

**Table 1:** **Adult patient characteristics at enrollment into HIV Care, by enrollment year**

	**Total**		**2006-2007**		**2008-2009**		**2010-2011**		**p-value** ^**1**^
	**n = 93,418**	**%** ^**2**^	**n = 34,528**	**%** ^**2**^	**n = 35,242**	**%** ^**2**^	**n = 23,648**	**%** ^**2**^	
**Age category**									<0.0001
15-24 yrs	14,997	16.1	5,361	15.5	5,687	16.1	3,949	16.7	
25-39 yrs	57,160	61.2	21,519	62.3	21,532	61.1	14,109	59.7	
40-49 yrs	14,675	15.7	5,490	15. 9	5,438	15.4	3,747	15.8	
50+ yrs	6,586	7.1	2,158	6.3	2,585	7.3	1,843	7.8	
**Sex**									0.0002
Male	36,906	39.5	13,917	40.3	13,857	39.3	9,132	38.6	
Female	56,512	60.5	20,611	59.7	21,385	60.7	14,516	61.4	
**Religion**									<0.0001
Orthodox	56,616	60.6	21,398	62.0	21,260	60.3	13,958	59.0	
Muslim	16,145	17.3	5,142	14.9	6,157	17.5	4,846	20.5	
Protestant	14,205	15.2	5,538	16.0	5,358	15.2	3,309	14.0	
Other/Unknown	6,452	6.9	2,450	6.8	2,467	6.6	1,535	6.1	
**Marital status**									<0.0001
Never Married	12,591	13.5	4,828	14.0	4,520	12.8	3,243	13.7	
Married/Living together	48,218	51.6	16,780	48.6	18,653	52.9	12,785	54.1	
Separated/Divorced	16,971	18.2	6,360	18.4	6,289	17.9	4,322	18.3	
Widowed	11,165	12.0	4,633	13.4	3,996	11.3	2,536	10.7	
Other/Unknown	4,473	4.8	1,927	5.6	1,784	5.1	762	3.2	
**Education**									<0.0001
No Education	27,547	29.5	9,309	27.0	10,697	30.4	7,541	31.9	
Primary	34,178	36.6	12,768	37.0	12,794	36.3	8,616	36.4	
Secondary	20,943	22.4	8,667	25.1	7,603	21.6	4,673	19.8	
Tertiary	5,565	6.0	1,606	4.7	2,118	6.0	1,841	7.8	
Other/Unknown	5,185	5.6	2,178	6.3	2,030	5.8	977	4.1	
**Point of entry into care**									<0.0001
PITC	34,566	37.0	9,538	27.6	14,425	40.9	10,603	44.8	
VCT	29,128	31.2	13,162	38.1	9,713	27.6	6,253	26.4	
Other Hospital/HC	12,930	13.8	6,418	18.6	4,142	11.8	2,370	10.0	
Community	1,143	1.2	483	1.4	370	1.0	290	1.2	
Unknown/missing	15,651	16.8	4,927	14.3	6,592	18.7	4,132	17.5	
**Transferred in**	1,607	1.7	526	1.5	535	1.5	546	2.3	<0.0001
**WHO Stage**									<0.0001
Stage I	23,979	26	6,891	20	9,767	28	7,321	31	
Stage II	16,595	18	5,272	15	6,655	19	4,668	20	
Stage III	33,072	35	13,299	39	12,023	34	7,750	33	
Stage IV	6,456	7	3,395	10	1,800	5	1,261	5	
Missing	13,316	14	5,671	16	4,997	14	2,648	11	
CD4+ cell count (cells/mm^3^)									
Median(IQR)	183 (87–342)		158 (76–293)		195 (93–360)		208 (98–376)		<0.0001
<100	19,392	21	8,017	23	6,814	19	4,561	19	
100-199	16,818	18	6,557	19	6,087	17	4,174	18	
200-350	15,077	16	5,006	14	5,809	16	4,262	18	
350+	16,364	18	4,612	13	6,650	19	5,102	22	
Missing	25,767	28	10,336	30	9,882	28	5,549	23	
TB screening at enrollment									<0.0001
Yes	54,033	57.8	11,275	32.7	23,505	66.7	19,253	81.4	
No/not recorded	39,385	42.2	23,253	67.3	11,737	33.3	4,395	18.6	
TB treatment at enrollment									<0.0001
Yes	8,445	9.0	3,091	9.0	2,969	8.4	2,385	10.1	
No/not recorded	84,973	91.0	31,437	91.0	32,273	91.6	21,263	89.9	
Cotrimoxazole at enrollment									<0.0001
Yes	59,652	63.9	20,032	58.0	22,870	64.9	16,750	70.8	
No/not recorded	33,766	36.1	14,496	42.0	12,372	35.1	6,898	29.2	
**Facility type**									<0.0001
Primary (11 sites)	4,844	5.2	1,316	3.8	1,934	5.5	1,594	6.7	
Secondary/tertiary (45 sites)	88,574	94.8	33,212	96.2	33,308	94.5	22,054	93.3	
**Setting**									<0.0001
Urban city (25 sites)	57,904	62.0	22,693	65.7	21,408	60.7	13,803	58.4	
Semi-urban (21 sites)	29,219	31.3	10,492	30.4	11,442	32.5	7,285	30.8	
Rural (10 sites)	6,295	6.7	1,343	3.9	2,392	6.8	2,560	10.8	
**Initiated ART**									
**Yes**	53,300	57.1	21,920	63.5	19,296	54.8	12,084	51.1	<0.0001
**No**	40,118	42.9	12,608	36.5	15,946	45.2	11,564	48.9	

^1^p-values based on chi-squared test for any difference across groups over time. see text for specific tests for trend.

^2^categorical percentages may not total exactly 100% due to roundoff errors.

**Table 2:** **Adult patient characteristics at ART initiation, by enrollment year**

	**Total**		**2006-2007**		**2008-2009**		**2010-2011**		**p-value** ^**1**^
	**n = 53,300**	**%** ^**2**^	**n = 21,920**	**%** ^**2**^	**n = 19,296**	**%** ^**2**^	**n = 12,084**	**%** ^**2**^	
**Age category**									<0.0001
15-24 yrs	6,040	11.3	2425	11.1	2191	11.4	1,424	11.8	
25-39 yrs	33,303	62.5	13901	63.4	12031	62.4	7,371	61.0	
40-49 yrs	9,722	18.2	4045	18.5	3453	17.9	2,224	18.4	
50+ yrs	4,235	8.0	1549	7.1	1621	8.4	1,065	8.8	
**Sex**									0.14
Male	22,302	41.8	9284	42.4	8004	41.5	5,014	41.5	
Female	30,998	58.2	12636	57.7	11292	58.5	7,070	58.5	
**WHO Stage at ART initiation**									<0.0001
Stage I	7,516	14.1	1830	8.3	3246	16.8	2,440	20.2	
Stage II	10,890	20.4	4002	18.3	4297	22.3	2,591	21.4	
Stage III	27,930	52.4	12403	56.6	9769	50.6	5,758	47.6	
Stage IV	5,894	11.1	3284	15.0	1580	8.2	1,030	8.5	
Missing	1,070	2.0	401	1.8	404	2.1	265	2.2	
**CD4+ cell count, cells/mm** ^**3**^ **at ART initiation**									
Median (IQR)	132 (68–197)		128 (66–192)		137 (71–201)		132 (65–202)		<0.0001
<100	18,227	34.2	7,816	35.7	6,247	32.4	4,164	34.5	
100-199	18,596	34.9	7,927	36.2	6,696	34.7	3,973	32.9	
200-350	10,829	20.3	4,063	18.5	4,121	21.4	2,645	21.9	
350+	935	1.8	409	1.9	309	1.6	217	1.8	
Missing	4,713	8.8	1,705	7.8	1,923	10.0	1,085	9.0	
**First ART regimen**									<0.0001
D4T-containing regimen	27,380	51.4	15,318	69.9	11,100	57.5	962	8.0	
AZT-containing regimen	13,509	25.4	5,733	26.2	5,159	26.7	2,617	21.7	
TDF-containing regimen	12,124	22.8	846	3.9	2,840	14.7	8,438	69.8	
Other	287	0.5	23	0.1	197	1.0	67	0.6	
**Facility type**									<0.0001
Primary (11 sites)	2,421	4.5	712	3.3	906	4.7	803	6.7	
Secondary/Tertiary (45 sites)	50,879	95.5	21,208	96.8	18,390	95.3	11,281	93.4	
**Setting**									
Urban city (25 sites)	33,491	62.8	14,498	66.1	11,705	60.7	7,288	60.3	<0.0001
Semi-urban (21 sites)	16,711	31.4	6,616	30.2	6,435	33.4	3,660	30.3	
Rural (10 sites)	3,098	5.8	806	3.7	1,156	6.0	1,136	9.4	
**CD4 testing performed on-site**									
Yes	44,353	83.2	18,699	85.3	15,889	82.3	9,765	80.8	<0.0001
No	8,947	16.8	3,221	14.7	3,407	17.7	2,319	19.2	

^1^p-values based on chi-squared test for any difference across groups over time. see text for specific tests for trend.

^2^ categorical percentages may not total exactly 100% due to roundoff errors.

Outcomes of patients in pre-ART care (pre-ART outcomes) were classified into the following mutually exclusive categories twelve months after enrollment: (1) initiating ART, (2) remaining in pre-ART care but not initiating ART, (3) transferring to another clinic before initiating ART, (4) recorded as dying before ART initiation, and (5) LTF before ART initiation. Patients were considered LTF before ART initiation if they were not recorded as dead, transferred, or initiating ART, and if they did not have a recorded visit for 12 months or more with no subsequent visit recorded in the database. Patients LTF were censored based on their date of last visit. We further divided patients LTF before ART initiation into those lost after only one (enrollment) visit and those lost after two or more pre-ART visits. Among patients ever initiating ART, we estimated the cumulative incidence of recorded death, LTF, and total attrition (recorded death or LTF) using Kaplan-Meier survival analysis. Patients were considered LTF after ART initiation when they were not recorded as dead or transferred, and if they did not have a recorded visit for 6 months or more. Individual-level factors associated with reported LTF and death after ART initiation were estimated using Cox Proportional Hazards models with robust sandwich estimates to account for within-clinic correlation. Covariates included in the model were a priori hypothesized to influence recorded death or LTF, and include: age, sex, religion, marital status, education level, WHO stage at ART initiation, CD4+ cell count at ART initiation, ART regimen, whether an individual switched regimens during follow-up, point of HIV testing (PITC, VCT, other hospitals/health centers, community referral, unknown/missing), facility type (primary vs. secondary/tertiary), location (urban, semi-urban, rural), and whether on-site CD4+ testing was provided. For analyses with recorded death as the outcome of interest, patients transferring to another facility or lost to follow-up were censored according to their date of last contact with the facility. For analyses with LTF as the outcome of interest, patients accrued follow-up time until a date of recorded death, transfer, LTF, or the end of the study follow-up period. Sensitivity analyses examined whether treating recorded death as a competing risk for LTF appreciably changed the results using methods for calculating a sub-distribution hazard outlined by Fine and Grey [17].

### Ethical approval

Use of anonymized patient-level data from health facilities was conducted as part of the Identifying Optimal Models of HIV Care and Treatment study protocol. All data were de-identified prior to analysis and the investigators had no access to identifiable patient information. Institutional Review Board (IRB) approval was obtained from the National Research Ethics Review Committee in Ethiopia; the study was designated non-human subjects research by the IRB Columbia University and the Center for Global Health at the US Centers for Disease Control and Prevention (CGH/CDC).

## Results

### Setting and patient characteristics at enrollment in care

A total of 93,418 adults (≥15 years of age) enrolled in HIV care at one of 56 health facilities were included in the analysis. Table 1 presents baseline characteristics of this population, stratified by year of enrollment. Twenty-five facilities (45%) were in urban, 21 (37%) in semi-urban and 10 (18%) in rural settings; 32 (57%) were regional public hospitals. Overall, 53,300 (57%) adults enrolled into HIV care eventually initiated ART.

Over 60% of the patient population was female. Most patients reported receiving either no formal education (29.5%) or education only up to the primary level (36.6%). Nearly two thirds (61%) of the patients were 25–39 years of age at enrollment with small changes in age distribution observed over time. Over half of the patients (52%) were married or living with their partners/spouses and 61% identified themselves as Orthodox Christian.

Nearly one-third (31%) of the patients enrolling in HIV care were referred from VCT testing points, 37% after being tested through PITC at inpatient, outpatient, tuberculosis clinics, and PMTCT points of service, 14% were tested at another hospital or health center, 1% through community-based testing and 17% had incomplete information on point of testing. The proportion of patients enrolled after testing at service points providing PITC increased substantially from 27.6% in 2006–2007 to 44.8 in 2010–2011 (p <0.0001 for trend over time), while the proportion identified through VCT declined from 38.1% in 2006–2007 to 26.4% in 2010–2011 (p <0.0001).

80,102 (86%) of patients had a recorded WHO stage at enrollment (24,979 (26%) stage I, 16,595 (18%) stage II, 33,072 (35%) stage III, 6,456 (7%) stage IV, and 13,316 (14%) without a recorded WHO stage). The proportion of patients enrolling with WHO stage III and IV decreased over time from 39% (stage III) and 10% (stage IV) in 2006–2007 to 33% (stage III) and 5% (stage IV) in 2010–2011 (p <0.0001 for trend). 67,651 (72%) patients had a recorded CD4+ cell count at enrollment while 25,767 (28%) were missing enrollment CD4+ results. Among those with a recorded enrollment CD4+ cell count, the median (IQR) cell count was 183 (87–342) cells/mm^3^. The median CD4+ cell count increased over time from 158 (76–293) cells/mm^3^ in 2006–2007 to 208 (98–376) cells/mm^3^ in 2010–2011 (p <0.0001 for trend). The proportion of patients enrolling in care with CD4+ cell count ≥350 cells/mm^3^ increased from 13% in 2006–2007 to 22% in 2010–2011 (p < 0.0001 for trend). Increases in median CD4+ cell count at enrollment were observed across all points of entry. Among patients referred from VCT, median (IQR) CD4+ cell count increased from 173 (86–319) cells/mm^3^ in 2006–2007 to 244 (120–425 cells/mm^3^) in 2010–2011. The increase in median CD4+ cell count at enrollment over time was smaller for other points of entry (PITC: 148 (70–205) cells/mm^3^ in 2006–2007, 187 (86–349) cells/mm^3^ in 2010–2011; Other hospitals/health centers: 143 (68–256) cells/mm^3^ in 2006–2007, 201 (94–360) cells/mm^3^ in 2010–2011; Community testing: 182 (94–355) cells/mm^3^ in 2006–2007, 271 (142–463) cells/mm^3^ in 2010–2011; Unknown/missing point of entry: 154 (69–291) cells/mm^3^ in 2006–2007, 211 (97–375) cells/mm^3^ in 2010–2011. Similarly, the percentage with WHO stage III/IV decreased over time for VCT (46.8% (2006–2007) to 31.3% (2010–2011), PITC (50.4% (2006–2007) to 44.1% (2010–2011), Other hospitals/health centers (53.2% (2006–2007) to 42.3% (2010–2011) and Unknown/missing point of testing (42.2% (2006–2007) to 30.2% (2010–2011), while it marginally increased for Community testing (46.4% (2006–2007) to 49.3% (2010–2011).

### Patient characteristics at ART initiation

Overall, 53,300 (57%) patients enrolled in care initiated ART during the period of observation. Table 2 presents patient characteristics at ART initiation. Patients initiating ART with WHO stage III or IV decreased from 56.6% (stage III) and 15% (stage IV) in 2006–2007 to 47.6% (stage III) and 8.5% (stage IV) in 2010–2011 (p <0.0001 for trend). Among the 48,587 (91%) patients initiating ART with a recorded CD4+ cell count at ART initiation, median (IQR) CD4+ cell count was 132 (68–197) cells/mm^3^ and remained stable over time.

Table 2 also highlights changes in the recommended first-line ART regimens over time, with stavudine (D4T)-containing regimens being replaced with tenovofir (TDF) in 2010. D4T-containing regimens (D4T/lamivudine(3TC)/nevirapine(NVP) or D4T/3TC/efavirenz(EFV) comprised 57.5% of the first ART regimens in 2008–2009, but only 8% in 2010–2011. Concurrently, use of TDF-containing regimens (TDF/3TC/NVP or TDF/3TC/EFV) rose from 14.7% in 2008–2009 to 69.8% in 2010–2011 (p <0.0001).

### Outcomes of patients one year after enrollment in HIV care

**Figure 1:**
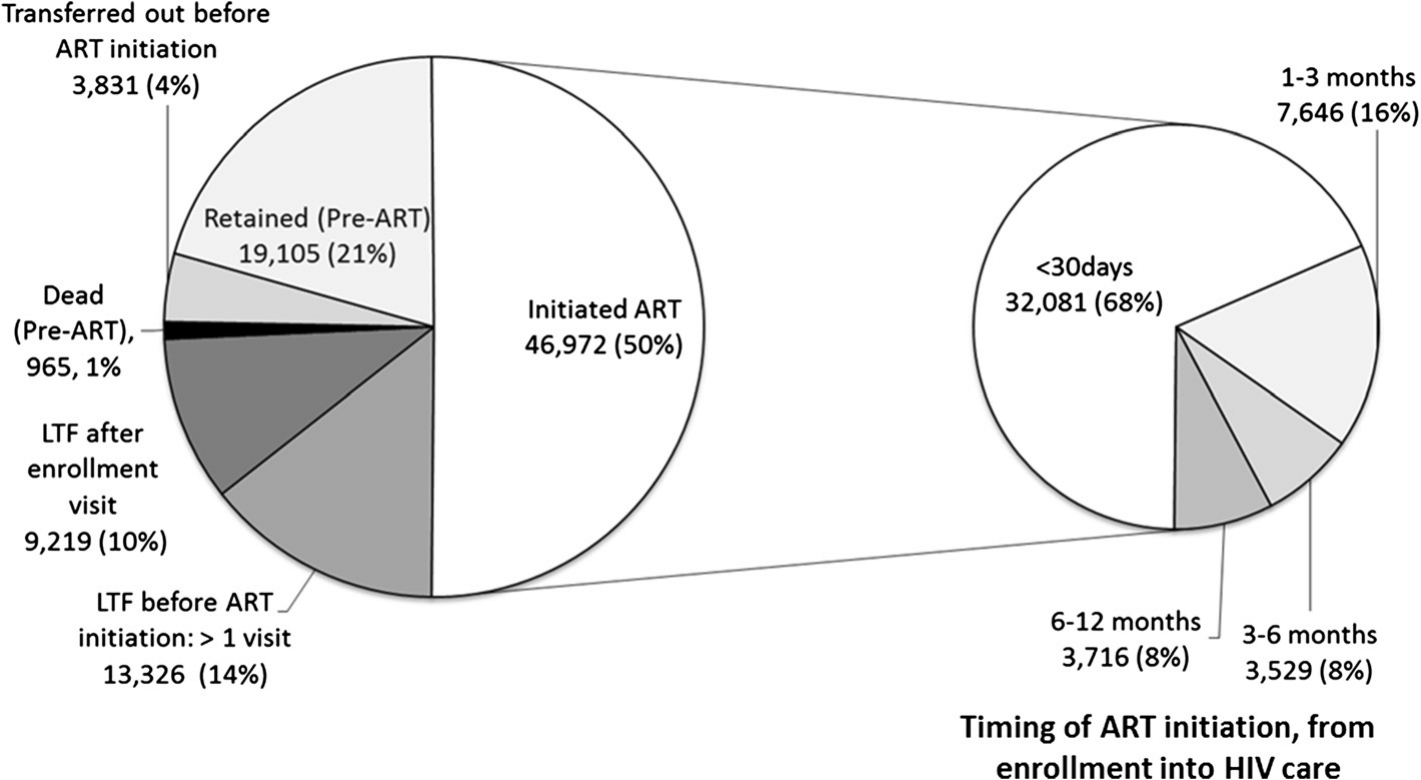
Patient outcomes 1 year after enrollment into HIV care (N = 93,418).

Figure 1 presents outcomes one year after enrollment into HIV care, and the subset of those initiating ART. Among all patients, 46,972 (50%) initiated ART within one year of enrollment. Among those not initiating ART within 1 year of enrollment, 13,869 (30%) did not initiate ART despite being ART eligible. 19,105 (21%) were retained in pre-ART care one year after enrollment, 3,831 (4%) transferred to another clinic before ART initiation, 965 (1%) were recorded deaths before ART initiation, and 22,545 (24%) became LTF before ART initiation. Among the 22,545 LTF, 9,219 (40.9%) were LTF after attending clinic for only the enrollment visit, while the remaining 13,326 (59.1%) were LTF after one or more follow-up visits. Among the 46,972 initiating ART within one year of enrollment into care, the majority (68%) initiated within 30 days. An additional 16% initiated within 3 months, 8% within 6 months, and 8% between 6 and 12 months after enrollment.

### Outcomes of patients after ART initiation

**Figure 2:**
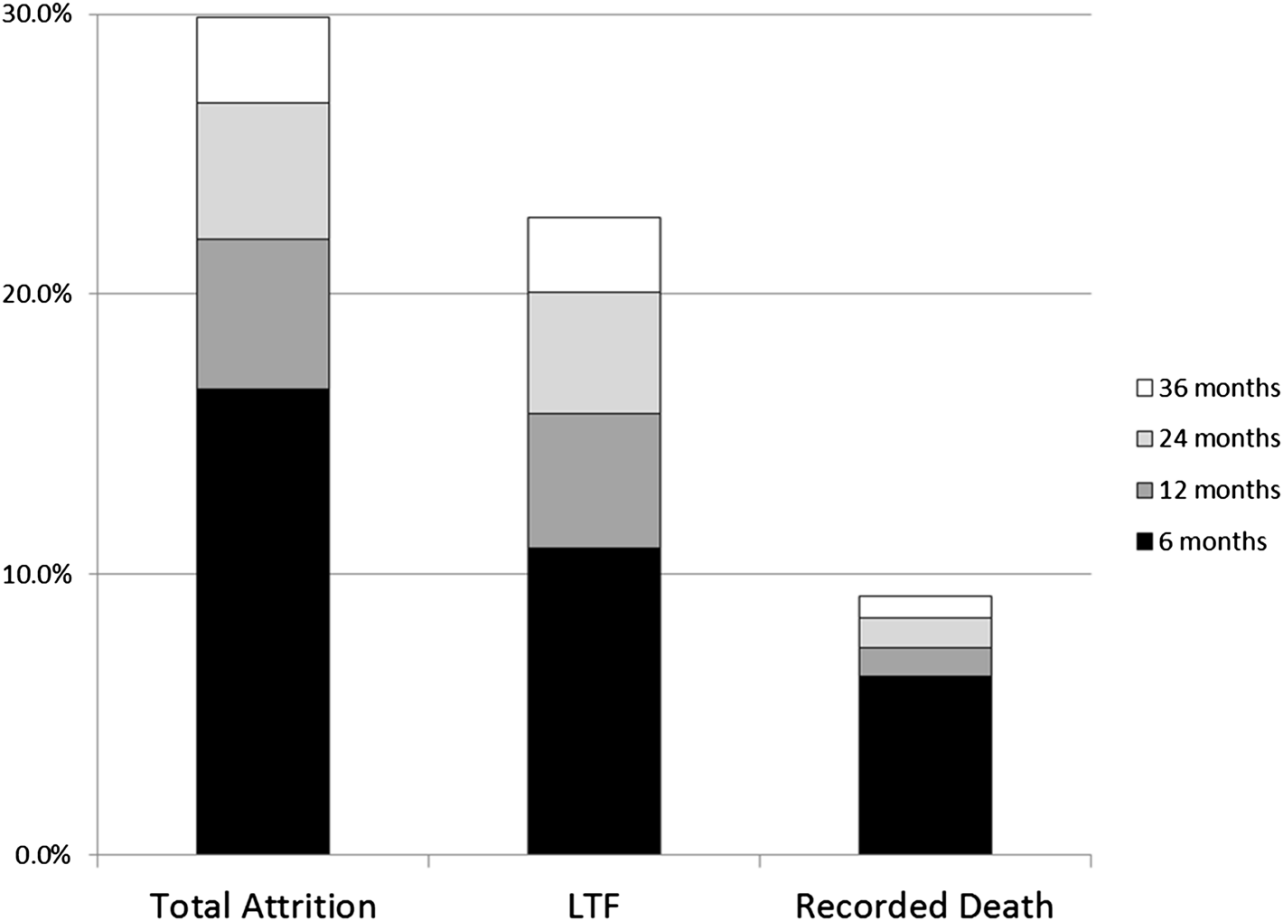
Cumulative incidence of recorded death, loss to follow-up, and total attrition (LTF or recorded death) after ART initiation, by time of event after ART initiation.

Figure 2 presents cumulative incidence of recorded deaths, LTF, and total attrition (LTF and recorded death) through 6, 12, 24, and 36 months after ART initiation. Overall, total attrition was 17% after 6 months, 22% after 12 months, 27% after 24 months, and 30% after 36 months. LTF was 11% at 6 months, 16% at 12 months, 21% at 24 months and 23% at 36 months indicating that nearly half of patients LTF after ART initiation were lost within in the first six months. Recorded death was 6% at 6 months, 7% at 12 months, 8% at 24 months, and 9% at 36 months, suggesting that approximately two-thirds of reported deaths after ART initiation occurred in the first 6 months.

**Figure 3:**
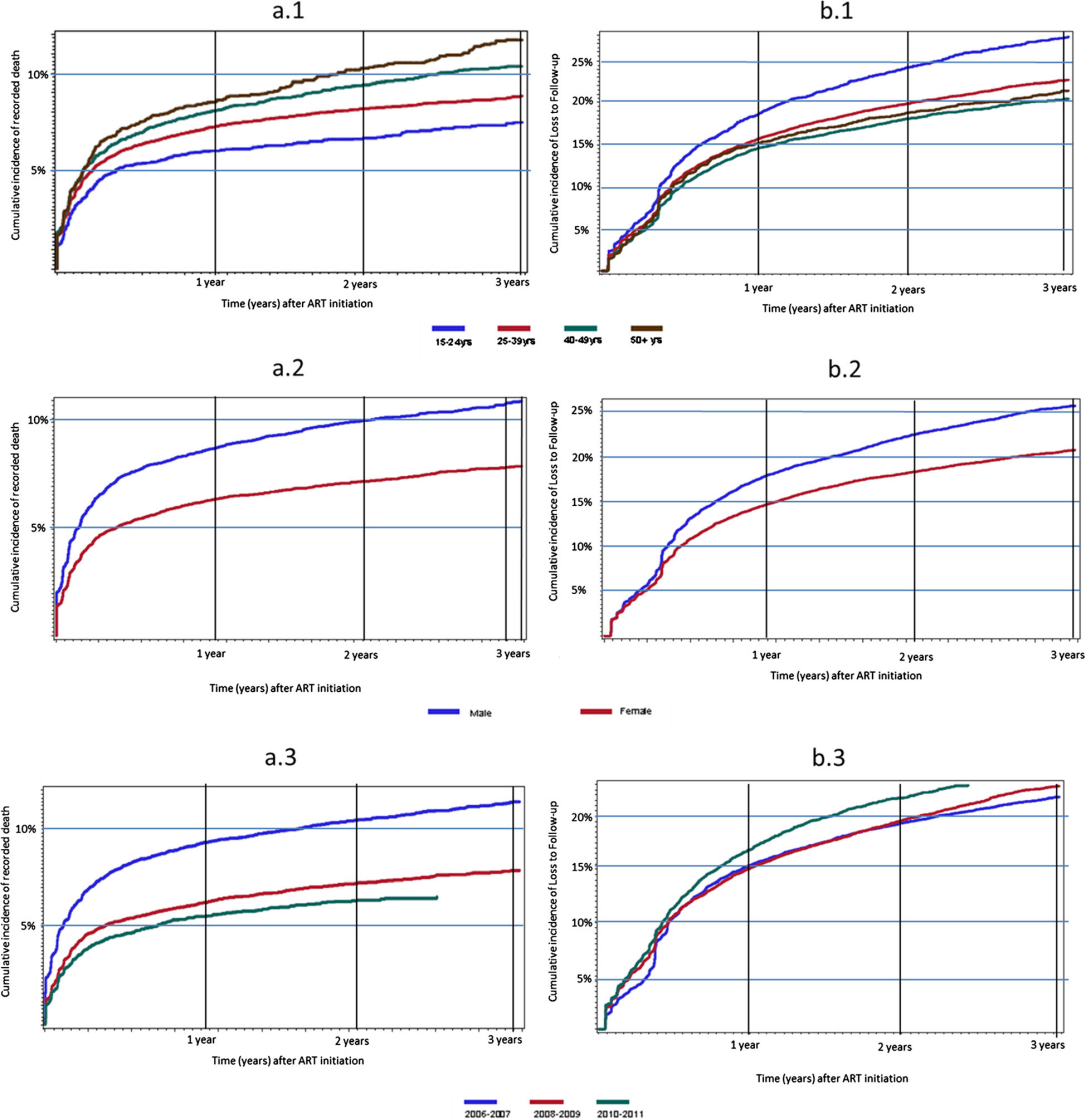
Cumulative incidence of recorded death (Figure a.1-a.3) and loss to follow-up (Figure b.1-b.3) after ART initiation. Figure a.1 and b.1: stratified by age at ART initiation. Figure a.2 and b.2: stratified by gender. Figure a.3 and b.3: stratified by year of ART initiation.

Cumulative incidence of recorded death (Figure 3a.1-a.3) and LTF (Figure 3b.1-b.3) after ART initiation are shown stratified by age (Figure 3a.1 and b.1), sex (Figure 3a.2 and b.2), and year of ART initiation (Figure 3c.1 and c.2). Young adults 15–24 years of age at ART initiation experienced lower cumulative incidence of recorded death (Figure 3a.1) but higher LTF (Figure 3b.1) than older age groups. Stratified by gender, males experienced higher recorded death and LTF after ART initiation compared with females. Cumulative incidence of recorded death decreased over time with adults initiating ART in 2006–2007 experiencing higher recorded deaths after ART initiation than adults initiating ART in 2010–2011. Loss to follow-up after ART initiation was slightly higher among patients initiating ART in 2010–2011 compared to patients initiating in 2006–2009.

Finally, we investigated individual-level factors associated with reported LTF and death after ART initiation (Table 3). Youth 15–24 years of age had the highest rates of LTF (aHR (50+ vs. 15–24): 0.67, 95% CI: 0.54-0.81; (40–49 vs. 15–24): 0.67, 95% CI: 0.60-0.75; (25–39 vs. 15–24): 0.77, 95% CI: 0.72-0.83), while adults 50 years and older had the highest rate of recorded death (aHR 1.61, 95% CI: 1.42-1.83; reference group = 15–24 years of age). Females had lower rates of LTF (aHR 0.73, 95% CI: 0.70-0.76) and slightly lower recorded death rates (aHR 0.90, 95% CI: 0.82-0.98) compared to males. Married individuals had the lowest rates of LTF and recorded deaths, and individuals reporting no formal education had higher LTF rates, but lower recorded death rates, compared to those reporting some education. Both LTF and recorded death were highest among individuals with WHO stage IV and those with CD4+ cell count <100 cells/mm^3^ at ART initiation. LTF increased with year of \ART initiation (2010–11 vs 2006–07 aHR 1.27, 95% CI:1.15-1.40), while recorded death decreased (201011 vs 2006-07 aHR 0.62, 95% CI: 0.50-0.75). Individuals referred from PITC (aHR 1.25, 95% CI: 1.20-1.31), other hospitals/health centers (aHR 1.12, 95% CI: 1.05-1.20), Community testing (aHR 1.18, 95% CI: 1.06-1.30) and Unknown/missing (aHR 1.10, 95% CI: 1.01-1.19) points of entry experienced higher LTF compared to those enrolling from VCT. Compared to VCT, recorded death after ART initiation was similar among patients enrolled from PITC (aHR 1.05, 95% CI: 0.92-1.19), Community testing (aHR 0.59, 95% CI: 0.30-1.14), slightly higher for Unknown/missing point of testing (aHR 1.14, 95% CI: 1.05-1.24) and slightly lower for other hospitals/health centers (aHR 0.83, 95% CI: 0.72-0.95).

**Table 3:** **Hazard ratio of LTF and death after ART initiation**

	**LTF**				**Recorded death**			
	**Crude**		**Adjusted**		**Crude**		**Adjusted**	
	**HR** ^**1**^	**95% CI**	**aHR** ^**1,2**^	**95% CI**	**HR** ^**1**^	**95% CI**	**aHR** ^**1,2**^	**95% CI**
**Age category**								
15-24 yrs	1	reference	1	reference	1	reference	1	reference
25-39 yrs	0.78	0.72-0.85	0.77	0.72-0.83	1.2	1.10-1.30	1.11	1.02-1.21
40-49 yrs	0.70	0.62-0.79	0.67	0.60-0.75	1.39	1.25-1.55	1.30	1.14-1.48
50+ yrs	0.74	0.60-0.91	0.67	0.54-0.81	1.54	1.38-1.73	1.61	1.42-1.83
**Sex**								
Male	1	reference	1	reference	1	reference	1	reference
Female	0.79	0.75-0.83	0.73	0.70-0.76	0.71	0.66-0.77	0.90	0.82-0.98
**Religion**								
Orthodox	1	reference	1	reference	1	reference	1	reference
Muslim	1.22	1.00-1.48	1.17	1.01-1.35	1.03	0.89-1.19	0.98	0.86-1.13
Protestant	0.89	0.72-1.11	0.99	0.88-1.10	0.84	0.70-1.00	0.77	0.69-0.86
Other/Unknown	1.20	1.01-1.43	0.91	0.80-1.03	1.15	0.87-1.53	0.88	0.70-1.10
**Marital status**								
Never Married	1	reference	1	reference	1	reference	1	reference
Married/Living together	0.63	0.58-0.67	0.67	0.63-0.71	0.8	0.68-0.95	0.84	0.75-0.95
Separated/Divorced	0.83	0.77-0.89	0.90	0.84-0.96	0.92	0.78-1.09	1.02	0.87-1.19
Widowed	0.68	0.63-0.73	0.78	0.71-0.86	0.76	0.66-0.88	0.92	0.79-1.06
Other/Unknown	1.04	0.78-1.39	0.97	0.80-1.03	1.12	0.73-1.72	1.08	0.88-1.33
**Education**								
No Education	1	reference	1	reference	1	reference	1	reference
Primary	0.74	0.70-0.79	0.71	0.66-0.75	1.23	1.16-1.29	1.27	1.18-1.36
Secondary	0.64	0.58-0.71	0.59	0.53-0.66	1.47	1.33-1.62	1.51	1.38-1.66
Tertiary	0.53	0.47-0.59	0.48	0.42-0.55	1.43	1.24-1.66	1.57	1.38-177
Other/Unknown	1.04	0.80-1.36	0.96	0.79-1.17	1.60	1.16-2.23	1.36	1.05-1.75
**WHO Stage at ART initiation**								
Stage I	1	reference	1	reference	1	reference	1	reference
Stage II	0.96	0.86-1.07	0.94	0.85-1.04	1.15	0.96-1.38	1.07	0.89-1.27
Stage III	1.30	1.15-1.47	1.19	1.07-1.32	2.06	1.70-2.49	1.68	1.41-1.99
Stage IV	1.80	1.48-2.18	1.60	1.38-1.85	4.25	3.26-5.52	2.98	2.32-3.84
Missing	1.28	1.12-1.47	1.18	1.07-1.33	2.3	1.69-3.11	1.66	1.22-2.24
**CD4+ cell count (cells/mm** ^**3**^ **) at ART initiation**								
<100	1.39	1.18-1.64	1.43	1.20-1.71	1.62	1.27-2.06	1.84	1.37-2.46
100-199	0.97	0.82-1.16	1.09	0.91-1.31	0.75	0.57-0.98	1.01	0.74-1.37
200-350	0.87	0.72-1.04	0.96	0.78-1.19	0.48	0.37-0.62	0.64	0.49-0.85
350+	1	reference	1	reference	1	reference	1	reference
Missing	1.36	1.09-1.70	1.46	1.20-1.77	1.67	1.26-2.21	1.81	1.36-2.41
**First ART regimen**								
D4T-containing regimen	1	reference	1	reference	1	reference	1	reference
AZT-containing regimen	0.86	0.75-0.98	0.76	0.67-0.85	0.75	0.64-0.88	0.79	0.69-0.91
TDF-containing regimen	0.92	0.81-1.04	0.66	0.59-0.74	0.60	0.51-0.70	0.74	0.64-0.86
Other	2.91	2.08-3.78	2.72	2.07-3.58	1.35	0.79-2.30	1.51	0.81-2.81
**Change of ART regimen**	0.26	0.21-0.32	0.25	0.19-0.31	0.48	0.42-0.56	0.41	0.35-0.49
**Enrollment year**								
2006-2007	1	reference	1	reference	1	reference	1	reference
2008-2009	1.05	0.96-1.14	1.05	0.95-1.14	0.58	0.49-0.69	0.69	0.60-0.78
2010-2011	1.15	1.00-1.33	1.27	1.15-1.40	0.67	0.62-0.74	0.62	0.50-0.75
**Point of entry**								
PITC	1.33	1.24-1.41	1.25	1.20-1.31	1.19	1.05-1.35	1.05	0.92-1.19
VCT	1	reference	1	reference	1	reference		
Other Hospital/HC	1.19	1.08-1.30	1.12	1.05-1.20	0.89	0.78-1.03	0.83	0.72-0.95
Community	1.42	1.23-1.63	1.18	1.06-1.30	0.56	0.24-1.31	0.59	0.30-1.14
Unknown/missing	1.20	1.07-1.34	1.10	1.01-1.19	1.08	0.90-1.30	1.14	1.05-1.24
**Facility type**								
Primary	0.67	0.49-0.92	0.86	0.60-1.24	1.03	0.63-1.69	1.28	0.77-2.14
Secondary/tertiary	1	reference	1	reference	1	reference	1	reference
**Setting**								
Urban city (25 sites)	1	reference	1	reference	1	reference	1	reference
Semi-urban (21 sites)	0.93	0.71-1.21	0.89	0.72-1.09	1.33	0.87-2.05	1.36	0.86-2.16
Rural(10 sites)	0.67	0.49-0.92	0.66	0.51-0.85	2.06	1.35-3.16	1.92	1.16-3.18
**CD4 testing on-site**	1.47	1.18-1.83	1.31	1.08-1.60	0.68	0.48-0.96	0.85	0.62-1.06

^1^Hazard ratios estimated using robust sandwich estimators for variance to account for within-clinic correlation.

^2^Adjusted for other variables in table.

Among facility-level characteristics considered (facility type, location, and availability of on-site CD4 testing), patients initiating ART in rural settings experienced less LTF compared to patients in urban settings (aHR 0.71, 95% CI: 0.54-0.95), with no observed difference in recorded death rates (aHR (semi-urban vs urban): 1.33 (0.91-1.93); rural vs urban: 0.86 (0.66-1.12).

In a sensitivity analysis examining whether treating recorded death as a competing risk for LTF appreciably altered our findings, the sub-distribution hazard competing risk model changed coefficients by an average of > 3%, suggesting trivial bias in censoring recorded deaths.

## Discussion

This analysis is the most comprehensive assessment to date of characteristics of the adult population enrolling in HIV care at PEPFAR-supported health facilities in Ethiopia during the period of ART scale-up and adoption of the opt out PITC approach. Ethiopia’s experience is unique in that it scaled up PITC in a low-prevalence setting without expanding guidelines for ART eligibility. In our analysis, we observed a substantial increase in the proportion of adults enrolling in HIV care after testing HIV-positive through PITC services (from 27.6% in 2006–2007 to 44.8% in 2010–2011). Routine HIV testing has been suggested to increase early diagnosis of HIV and improve access to HIV care [18], but other investigations have concluded that PITC is more likely to incidentally identify later-stage HIV positive individuals who have not previously sought out an HIV test through VCT [19]. In this analysis, increases in average CD4+ cell count, and lower average WHO stage, at enrollment into HIV care were observed over time across all points of testing, although the smallest increase occurred among those testing through PITC. Temporal trends toward increasing average CD4+ cell count, and decreasing average WHO stage, at enrollment have been observed elsewhere [20].

Enrollment of patients into HIV care at earlier disease stages seems to have reduced somewhat the proportion of those initiating ART with WHO stage III or IV disease. However, median CD4+ cell counts at ART initiation remained stable over time. This, coupled with unchanged guidelines based on WHO 2006 recommendations for ART initiation (CD4 cell count ≤ 200 cells/mm^3^) in Ethiopia during the time period covered [21], suggests that identification of HIV positive patients at an earlier stage in disease progression will not guarantee earlier ART initiation. Countries which have expanded guidelines for ART initiation have seen modest increases in median CD4+ cell counts at ART initiation over time [22]. Ethiopia has recently adopted WHO 2013 guidelines for initiating patients with a CD4+ cell count < 500 cells/mm^3^ and future analyses can investigate the impact of guideline switching on median CD4+ cell counts at ART initiation.

Nearly a quarter of adults enrolling into HIV care were LTF before ART initiation. This finding is similar to results reported by Mulissa et al. from one hospital in Ethiopia [7], and lower than estimates from a multi-country study in Kenya, Mozambique, Rwanda, and Tanzania [23] and from a sub-district in KwaZulu-Natal, South Africa [24]. Reasons for LTF before ART initiation are myriad but likely include lack of documentation of transfers and deaths along with true disengagement from care. In our study, LTF was higher among males than females, which is similar to reports from other settings [25]. Pre-ART retention is important to optimize health outcomes as it enables patients to be consistently monitored and promptly initiated on ART when eligible. The high attrition before ART initiation in this population, while lower than observed in other settings, highlights an important area of potential intervention in the HIV care cascade. Better patient tracing procedures, better understanding of loss to follow-up and earlier initiation of ART to reduce mortality have been recommended to improve retention [26-28].

After ART initiation, most attrition occurred within 6 months, suggesting that the initial time period after a patient initiates ART may be the most critical point in which to focus efforts aimed at improving retention. Early patient losses have also been reported from other cohorts [29,30]. With a better understanding of the reasons for LTF after ART initiation, interventions can be designed that improve treatment retention and ultimately, patient outcomes.

Twelve-month recorded mortality after ART initiation was 7.4% in our population. This is similar to a study from Mozambique using routinely-collected service delivery data [31], and somewhat lower than reported from other countries in sub-Saharan Africa [32]. Our measure of recorded mortality is an underestimate of the true mortality as we did not actively ascertain death records. LTF is comprised of individuals truly defaulting from care, in addition to undocumented transfers and unascertained deaths. We expect that a large but unknown proportion of the patients classified as LTF (16% at 12 months among adults initiating ART in our analysis) were actually unascertained deaths, and our analysis found that markers of poor immunologic health at ART initiation (low CD4+ cell count and WHO stage III/IV) were associated with increased hazard of LTF. For example, if we were to assume that approximately 33% of the patients LTF were actually unreported deaths [33], our mortality estimate would increase to 10.6%, similar to pooled estimates reported from studies in sub-Saharan Africa [32]. Over time, recorded mortality after ART initiation decreased substantially, with individuals initiating ART in 2010–2011 having 60% lower hazard of recorded death compared to individuals initiating ART in 2006–2007. This decrease was substantially larger than the increase in LTF observed between the same time periods, suggesting that differential ascertainment of deaths does not explain the improvement in mortality over time.

The strengths and weaknesses of this study both arise from the data source. We used data routinely collected as part of clinical HIV care in Ethiopia, rather than data collected as part of a research study. Clinical data often has more missing information than that collected as part of a research study, as evidenced by the high levels of missing data on CD4+ cell counts at enrollment in HIV care. In addition, we relied on death recorded as part of routine clinical care and did not independently verify vital status. Additionally, a large proportion of patients were classified as having a point of HIV testing at another hospital or health center (14%) or had missing information on point of testing (17%). For these patients, we are unable to ascertain whether the testing was through PITC or VCT. However, utilization of routinely-collected data has enabled us to report on a very large population of individuals seeking HIV care in Ethiopia. Our study population comprised 53,300 adults initiating ART between 2006 and 2011 in Ethiopia. UNAIDS estimates of the total number of individuals currently receiving ART in Ethiopia through the end of 2011 to be approximately 265,000 [34], suggesting that approximately 20% of adults receiving ART in Ethiopia are included in our study. Consequently, our assessment provides a more representative sample of patients and outcomes from the types of clinics where the majority of HIV-positive individuals in Ethiopia are seeking HIV care.

## Conclusions

These Ethiopian health facilities have shown earlier identification and engagement in care of HIV-positive individuals over time across all points of HIV testing. Patients at the HIV clinics included in this analysis were enrolled in HIV services at higher median CD4+ cell counts and lower WHO stage over time, and fewer patients initiated ART with advanced WHO stage. Pre-ART retention, prompt initiation of eligible persons on ART, and retention after ART initiation (particularly in the first 6 months) remain major challenges.
